# Report from the ‘*One Health*’ 9th Tick and Tick-Borne Pathogen Conference and the 1st Asia-Pacific Rickettsia Conference, Cairns, Australia, 27 August–1 September 2017

**DOI:** 10.3390/vetsci5040085

**Published:** 2018-10-02

**Authors:** Ala Tabor, Manuel Rodriguez Valle

**Affiliations:** Centre for Animal Science, Queensland Alliance for Agriculture & Food Innovation, The University of Queensland, Brisbane 4072, Australia

**Keywords:** *One Health*, ticks, tick-borne diseases, conference report

## Abstract

The 9th Tick and Tick-borne Pathogen (TTP9) Conference was held in conjunction with the first Asia Pacific Rickettsia Conference (APRC1) in Cairns, Australia from 27 August until 1 September in 2017. This MDPI Veterinary Sciences Special Issue has been dedicated to selected veterinary science articles from the conference associated with the control of animal diseases in the context of ticks and tick-borne pathogens, including Rickettsia species. The articles presented in this Special Issue include novel developments for the future control of ticks and tick-borne diseases. This editorial describes the meeting content, the plenaries, the TTP awards, the MDPI Veterinary Science Special Issue articles, and serves as a legacy report for TTP9APRC1.

## 1. Background

**Figure 1:**
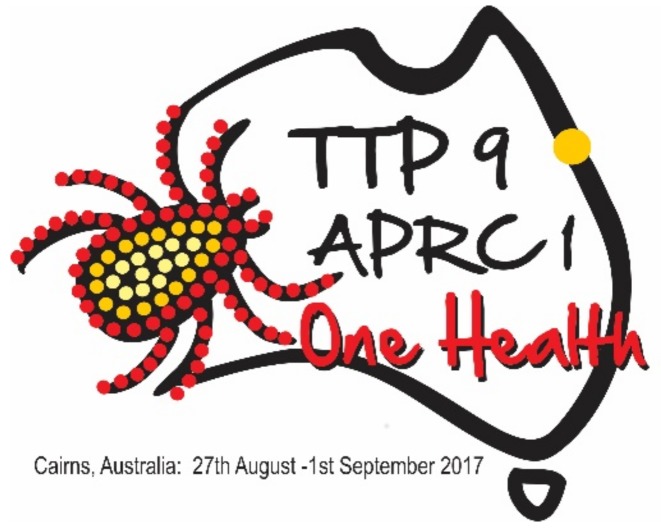
The TTP9APRC1 conference logo styled as the map of Australia highlighting the location of Cairns. The tick is depicted with an Australian indigenous art style with the dots representing vectored pathogens.

Tick and Tick-borne Pathogen (TTP9) conferences have been held around the globe since 1992 (See Table 1). The conferences have in a few instances partnered with other organisations, such as TTP8 with the 12th Biennial Society for Tropical Veterinary Medicine (STVM) in 2014 (South Africa), and TTP9 with the inaugural Asia Pacific Rickettsia Conference (APRC1) in 2017, see Figure 1 for the conference logo. The mixture of medical, veterinary and wildlife researchers at TTP9APRC1 was quite a unique blend of conference delegates under the banner of ‘*One Health*’. TTP conferences are not underpinned by a professional society, thus, partnerships can increase sponsorship support and introduce a higher scientific and international content to the meeting. TTP10 will be held in Romania as the voted winner of bids from four countries in total with tentative dates as 24–28 August 2020.

## 2. Invited Plenaries

The conference invited 8 excellent plenary speakers with four selected by a TTP International conference alumni group and four chosen by the APRC1 sub-committee. These speakers, their institutions, and countries are summarised in Table 2. 

**Figure 2:**
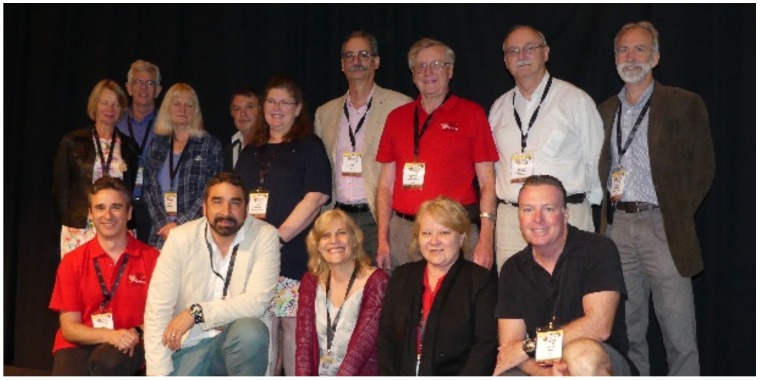
Fourteen members of the total 28 members of the Scientific Program Committee including the TTP9APRC1 Australian Local Organizing Committee Conference Convenors. First row: Prof. John Stenos (APRC1 Convenor), Prof. Manuel Rodriguez Valle (TTP9 Convenor), Prof. Monica Florin-Christensen (Argentina), Prof. Ala Tabor (TTP Convenor), Dr Robert Miller (USA). Second row: Prof. Pat Nuttall (UK, also plenary), Dr Peter Rolls (Australia), Prof. Ulrike Munderloh (USA, also plenary) Prof. Petr Kopáček (Czech Replubic), Prof. Kelly Brayton (USA), Prof. José de la Fuente (Spain), Prof. Stephen Graves (APRC1 Convenor), Prof. Gerhard Dobler (Germany), Dr Allen Richards (USA).

The program at the conference highlighted two plenary speakers daily which were usually followed by two concurrent sessions (except Friday) with a further 124 oral presentations presented over the four days. The conference also hosted approximately 125 posters which had dedicated poster viewings over two hours each on two consecutive days. Oral and poster abstracts were reviewed by an international expert panel who subsequently served as session Chairs at the conference, Figure 2. Scientific Program members absent in the photograph in Figure 2 include: Miles Beaman (Australia), Stuart Blacksell (USA), Richard Bradbury (USA), Lidia Chitimia-Dobler (Germany), Constantin Constantinoiu (Australia), Augustin Estrada-Peña (Spain), Roy Hall (Australia), Nicholas Jonsson (UK), Kersh Gilbert (USA), Christine Maritz-Olivier (South Africa), Jere McBride (Australia), John McBride (Australia), Robert Norton (Australia), and Jenny Robson (Australia).

**Figure 3:**
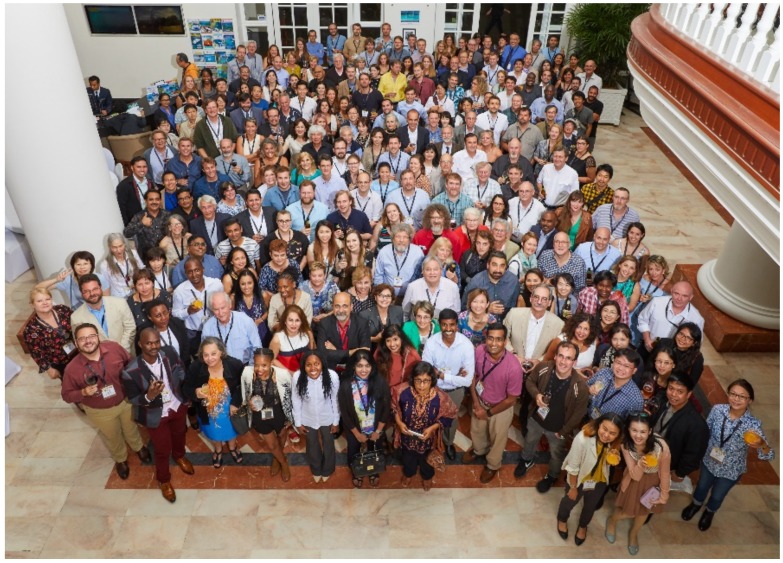
TTP9APRC1 2017 delegate group photo at the foyer of the Pullman Hotel (conference venue), Cairns, Australia.

The conference was attended by 240 delegates from and an impressive 38 different countries world-wide including: Argentina, Australia, Austria, Belgium, Bhutan, Brazil, Cambodia, Cuba, Czech Republic, France, Germany, Hungary, India, Iran, Italy, Japan, Laos, Malaysia, Mexico, Netherlands, New Caledonia, New Zealand, Nigeria, Norway, Romania, Slovakia, South Africa, South Korea, Spain, Switzerland, Taiwan, Thailand, Turkey, United Kingdom, United States of America, Vietnam, and Zambia. The TTP9APRC1 conference delegate group photo is presented in Figure 3. 

## 3. TTP9 Awards

**Figure 4:**
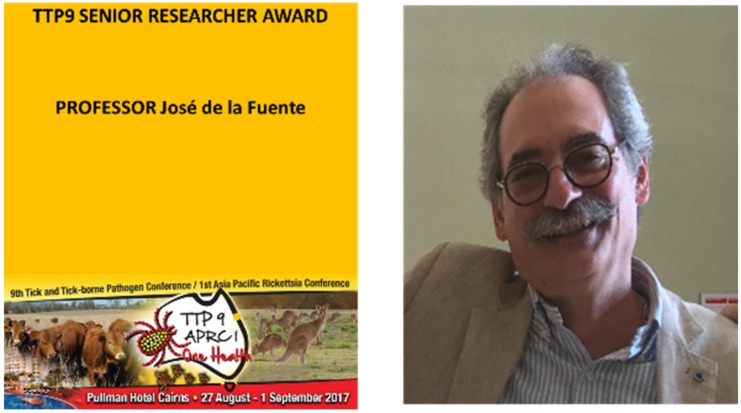
TTP9 Senior Researcher awardee.

TTP9 and APRC1 separately had awards for students and TTP9 continued the tradition for an award for an ‘*Outstanding Contributor to the Field of Tick and Tick-Borne Pathogens*’. TTP9’s Senior Researcher Award was awarded to Professor José de la Fuente of SaBio, Instituto de Investigación en Recursos Cinegéticos, Ciudad Real, SPAIN and Adjunct with Oklahoma State University, USA. He presented a plenary entitled ‘*Controlling ticks and tick-borne diseases…looking forward*’ which described his vision for future research themes for ticks and tick-borne diseases, see Figure 4.

The TTP9 young scientist awards were shared by two PhD students: Ms. Ronel Pienaar from the Onderstepoort Veterinary Institute and the University of Pretoria, South Africa, and Mr. Kodai Kusakisako from Kagoshima University and Yamaguchi University, Japan. The titles of their presentations were ‘The salivary gland transcriptome of *Rhipicephalus evertsi evertsi*, causative agent of Spring lamb paralysis’ and ‘Peroxiredoxins are important for blood feeding and reproduction through the regulation of hydrogen peroxide concentrations in the hard tick *Haemaphysalis longicornis*’, respectively. We congratulate all awardees. 

## 4. Special Issue in MDPI *Veterinary Sciences*

For invitations to this Special Issue we focused on obtaining a wide variety of articles of interest within the specific field of Veterinary Science, see Table 3. In many instances, junior post-graduate students or early career post-doctorate scientists were the first authors representing senior authors who presented at the TTP9APRC1 conference. This is an excellent outcome for the Special Issue as mentoring students to publish is a high priority for all! 

## 5. Conclusions

The conference’s scientific outcomes include the establishment of an *Ixodes* spp. genome International Consortium which aims to sequence tick *Ixodes* spp. globally to ultimately develop tools currently not available for studying tick populations including single nucleotide polymorphism genotyping. *Ixodes* spp. are known to bite mammals and, thus, impact human health. Sequencing technologies now available will enable the sequencing of complicated large tick genomes relevant to human and animal health. Other scientific outcomes include the networking of scientists from very diverse backgrounds which led to new partnerships across medical and veterinary fields. This Special Issue provided opportunities for veterinary presentations from the conference to be published. The articles selected here demonstrate the application of ‘omic’ technologies to study different livestock diseases, as well as transmission studies of a sheep pathogen which can cause infections in humans. The latter an excellent example addressing the ‘*One Health*’ theme of the conference. 

The TTP9 Convenors/Editors are very grateful to the students and lead scientists for their contributions to this Special Issue entitled: “*One Health*—9th Tick and Tick-borne Pathogen Conference and 1st Asia Pacific Rickettsia Conference” which can be found at this website: http://www.mdpi.com/journal/vetsci/special_issues/TTP9. We look forward to future TTP conferences, and the sharing of knowledge in this specialised field of ‘*One Health*’ associated with ticks and tick-borne diseases. We also thank the scientific program committee members for their contribution to the development of a great conference program! 

## Figures and Tables

**Table 1:** List of Tick and Tick-borne Pathogen conferences.

TTP	Year	Location	Country
TTP1	1992	Minnesota	USA
TTP2	1995	Kruger National Park	South Africa
TTP3	1999	Tatra Mountains	Slovakia
TTP4	2002	Banff	Canada
TTP5	2005	Neuchatel	Switzerland
TTP6	2008	Buenos Aires	Argentina
TTP7	2011	Zaragoza	Spain
TTP8	2014	Cape Town	South Africa
TTP9	2017	Cairns	Australia
TTP10	2020	Danube Delta	Romania

**Table 2:** TTP9APRC1 plenary speakers, their institutions, countries, and plenary titles.

Plenary	Institute	Plenary title	Country
Prof. Stephen Barker	The University of Queensland	‘When and where did ticks evolve, with notes on the ticks of Cairns and the Wet-Tropics of Australia and New Guinea’	Australia
Prof. Pierre-Edouard Fournier	Institut Hospitalo-Universitaire (IHU) Méditerranée Infection	‘Genomics of Rickettsia species’	France
Prof. Catherine A. Hill	Purdue University	‘Combating tick-borne diseases on a global scale: a roadmap for tick research in the genomics era’	USA
Prof. Peter Irwin	Murdoch University	‘The microbes of Australian ticks: their diversity and zoonotic potential’	Australia
Prof. Ulrike Munderloh	University of Minnesota	‘Mutational analysis of gene function in the Anaplasmataceae’	USA
Prof. Pat Nuttall	University of Oxford	‘The wonders of tick saliva’	UK
Prof. Daniel Paris	Swiss Tropical and Public Health Institute	‘Recent discoveries in scrub typhus research’	Switzerland
Dr Carlos E Suarez	US Department of Agriculture	‘Transfection approaches for developing novel vaccines against tick and tick borne diseases’	USA

**Table 3:** 

First Author	Title of Article	Country
Mariano E. Ascencio, et al. [1]	‘Cysteine protease C1 paralog profiles correspond with phylogenetic lineages of pathogenic piroplasmids’	Argentina
Diego Josimar Hernández-Silva, et al. [2]	‘Immunomolecular characterization of MIC-1, a novel antigen in *Babesia bigemina*, which contains conserved and immunodominant B cell epitopes that induce neutralizing antibodies’	Mexico
Paidashe Hove, et al. [3]	‘Detection and characterization of *Anaplasma marginale* and *A. centrale* in Africa’ (Review)	South Africa
José Manuel Jaramillo- Ortiz, et al. [4]	‘Development of an Indirect ELISA based on a recombinant chimeric protein for the detection of antibodies against bovine babesiosis’	Argentina
Thomas P. Karbanowicz, et al. [5]	‘Comparison of protein gut samples from *Rhipicephalus* spp. using a crude and an innovative preparation method for proteome analysis’	Australia
Brenda Leal, et al. [6]	‘Population dynamics of off-host *Rhipicephalus (Boophilus) microplus* (Acari: Ixodidae) larvae in response to habitat and seasonality in south Texas’	USA
Paulo H. C. Lima, et al. [7]	‘The *Nothoaspis amazoniensis* complete mitogenome: A comparative and phylogenetic analysis’	Brazil
Ronel Pienaar, et al. [8]	‘Tick paralysis: Solving an Enigma’ (Review)	South Africa
Snorre Stuen, et al. [9]	‘Intrauterine transmission of *Anaplasma phagocytophilum* in persistently infected lambs’	Norway
